# ZEB2/TWIST1/PRMT5/NuRD Multicomplex Contributes to the Epigenetic Regulation of EMT and Metastasis in Colorectal Carcinoma

**DOI:** 10.3390/cancers14143426

**Published:** 2022-07-14

**Authors:** Yayuan Zheng, Mingrui Dai, Yue Dong, Hanqiao Yu, Tianfu Liu, Xuejian Feng, Bin Yu, Haihong Zhang, Jiaxin Wu, Wei Kong, Xianghui Yu, Hui Wu

**Affiliations:** 1 National Engineering Laboratory for AIDS Vaccine, School of Life Sciences, Jilin University, Changchun 130012, China; zhengyayuan2022@163.com (Y.Z.); daimr21@mails.jlu.edu.cn (M.D.); dongy2013@163.com (Y.D.); 13910351667@163.com (H.Y.); liutianfu0131@163.com (T.L.); fengxj21@mails.jlu.edu.cn (X.F.); yubin@jlu.edu.cn (B.Y.); zhanghh@jlu.edu.cn (H.Z.); wujiaxin@jlu.edu.cn (J.W.); weikong@jlu.edu.cn (W.K.); 2 Key Laboratory for Molecular Enzymology and Engineering, The Ministry of Education, School of Life Sciences, Jilin University, Changchun 130012, China

**Keywords:** colorectal cancer, ZEB2, TWIST1, E-cadherin, epithelial-mesenchymal transition, tumor metastasis, PRMT5, NuRD

## Abstract

**Simple Summary:**

The epithelial-mesenchymal transition (EMT) program plays a central role in the metastasis of patients with colorectal carcinoma (CRC). However, few studies have investigated the dominant regulatory factors and underlying mechanisms during the EMT process in CRC metastasis. Here we have characterized a novel transcriptionally repressive complex, ZEB2/TWIST1/PRMT5/NuRD, which epigenetically silences E-cadherin, leading to the invasion and metastasis of CRC cells. Our work provides a comprehensive understanding of the epigenetic mechanisms by which the key EMT metastasis driver, E-cadherin, is regulated in CRC, thereby suggesting PRMT5 and HDAC2 inhibitors as potentially therapeutic agents in CRC therapy.

**Abstract:**

(1) Background: The EMT plays a crucial role in tumor metastasis, which is the major cause for colorectal carcinoma-related mortality. However, the underlying regulators and mechanisms of EMT in CRC metastasis are still poorly understood; (2) Methods: The transcriptional regulators of EMT in CRC and their functions were examined using RT2212PCR, Western blotting, and luciferase reporter assay. The components of ZEB2/TWIST1 complex and their mutual interactions were identified via affinity purification, mass spectrometry, co-immunoprecipitation, and pull-down experiments. The functional mechanisms of ZEB2/TWIST1/PRMT5/NuRD axis were determined by chromatin immunoprecipitation and luciferase reporter assay. The contribution of ZEB2/TWIST1/PRMT5/NuRD complex in the CRC metastasis was investigated using wound healing, transwell assay, and in vivo xenograft mouse model; (3) Results: We found that ZEB2 and TWIST1 were both significantly upregulated in CRC tissues and EMT of CRC cells. ZEB2 could recruit TWIST1 to the E-cadherin promoter and synergistically repressed its transcription. In addition, ZEB2 physically interacted with TWIST1, PRMT5, and the nucleosome remodeling and deacetylase (NuRD) complex to form a novel repressive multicomplex, leading to epigenetic silencing of E-cadherin in CRC cells. Notably, the combined inhibition of ZEB2 and TWIST1 and epigenetic inhibition markedly reduced CRC metastasis in mice; (4) Conclusions: We revealed for the first time that ZEB2 could recruit TWIST1, PRMT5, and NuRD to form a repressive multicomplex and epigenetically suppresses the transcription of E-cadherin, thereby inducing the EMT process and metastasis in CRC. Our results also confirmed the therapeutic potential of epigenetic inhibitors in CRC.

## 1. Introduction

Until now, 35% to 45% of patients with CRC still develop local relapse or distant metastasis [1,2], even though significant advances in colorectal carcinoma (CRC) therapies, including surgery, chemotherapy, and radiotherapy, have increased the overall survival rates in the early stages of the disease. Nevertheless, there are no effective therapies for metastatic CRC patients, and the 5-year survival rate is less than 10%. Increasing studies have demonstrated that the EMT program plays a central role in the metastasis in patients with CRC [3]. The EMT process is a cellular program in which the non-motile epithelial cells are transformed to mesenchymal cells with invasive behavior [4]. The markers of EMT are the downregulation of epithelial markers, specifically E-cadherin, and the acquisition of mesenchymal markers, such as N-cadherin and vimentin. Several studies have showed that reduced levels of E-cadherin are significantly associated with lymph node metastasis and poor prognosis in patients with CRC [5,6]. Toiyama et al. showed that the upregulation of vimentin indicated lymph node metastases and a worse prognosis [7]. Therefore, understanding the basic molecular mechanisms underlying the EMT progress during tumor metastasis is invaluable to the development of effective therapeutic approaches for patients with metastatic CRC. The EMT process is initially induced by three core families of transcriptional regulators, including ZEB, Snail, and TWIST1, which in turn induce the transcriptional inhibition of E-cadherin [8]. These EMT transcriptional repressors often function independently or in combination to promote EMT and tumor metastasis. For instance, high expression of TWIST1 is confined to colorectal tumor tissues and correlates with lymph node metastasis and poor survival rate [9]. ZEB2 also promotes tumor metastasis and is associated with a poor prognosis in patients with CRC [10]. In addition, co-expression of TWIST1 and ZEB2 was observed in patients with oral squamous cell carcinoma and was significantly associated with poor survival [11]. Larriba et al. have found that Snail2 cooperates with Snail1 to repress the vitamin D receptor and block the induction of E-cadherin in colon cancer [12]. However, few studies have investigated the dominant regulatory factors and underlying mechanisms during the EMT process in CRC metastasis. In addition, recent studies have revealed that EMT transcriptional factors cooperate with a variety of histone-modifying enzymes to repress the E-cadherin promoter at the epigenetic level [13]. Multiple histone lysine methyltransferases, such as G9a and EZH2, have been shown to be involved in the EMT and metastasis of various cancers [14,15,16]. In contrast, the function of histone arginine methyltransferase in the tumor metastasis is not as well defined. Protein arginine methyltransferase 5 (PRMT5) can silence genes through the symmetric demethylation of H4R3 and H3R8 histones. It plays important roles in cell invasion and metastasis by interacting with several epigenetic regulators and regulating the transcription of target genes [17,18,19]. The PRMT5/WDR77 complex cooperates with PHF1, a novel reader for the H4R3 histone symmetric demethylation, to promote cell proliferation, invasion, and tumorigenesis [20]. Recently, Gao and colleagues have shown that PRMT5 associated with Snail and NuRD complex to induce methylation and repression of E-cadherin in cervical cancer [21]. Nevertheless, the roles and molecular mechanism of PRMT5 in the metastasis of CRC remain poorly understood.

Histone deacetylation also plays an important role in transcriptional repression and is highly associated with malignancy progression [22]. Histone deacetylases (HDAC), which catalyze the removal of acetyl residues from inactive chromatin, are part of multimeric complexes, including co-activators or co-repressors, which are recruited to the E-cadherin promoter. Snail1 directly recruits HDAC1, HDAC2, and the co-repressor Sin3A to the CDH1 promoter to repress its expression by histone deacetylation [22]. HDAC1 and HDAC2 are recruited by ZEB1 to synergistically downregulate E-cadherin expression in pancreatic cancer [23]. The NuRD complex is a large macromolecular complex coupling the histone deacetylases (HDAC1/HDAC2) and the chromatin remodeling ATPase Mi-2. In addition to having two subunits with enzymatic activity, the Mi-2/NuRD complex also contains several other proteins, including MTA2, RbAp48, and MBD proteins. NuRD-mediated epigenetic regulation has been shown to play a significant role in promoting EMT and tumor metastasis. TWIST1 interacts with several components of the NuRD complex to recruit it to the E-cadherin promoter [24]. Snail can also form a repressive complex with NuRD and PRMT5 and contribute to E-cadherin repression in cervical cancer [21]. However, the functions and regulatory mechanisms of NuRD during EMT and metastasis of CRC are still not well defined. 

In the current study, we showed that ZEB2 and TWIST1 were both significantly upregulated in the EMT process of CRC cells and CRC patient tissues. ZEB2 recruited TWIST1 to the E-cadherin promoter and synergistically repressed transcription. In addition, ZEB2 physically interacted with TWIST1, PRMT5, and NuRD complex to form a novel transcriptionally repressive complex, mediating H3R4 methylation and H3K56 deacetylation of the E-cadherin promoter in CRC cells. Strikingly, ZEB2 and TWIST1 downregulation significantly inhibited CRC metastasis in mice models, and epigenetic inhibitors reduced it. Thus, our study provides mechanistic insights into a novel epigenetic regulatory network during the EMT process and metastasis of CRC, suggesting the potential therapeutic role of epigenetic inhibitors in CRC treatment.

## 2. Materials and Methods

### 2.1. Tissue Samples

Forty-nine paired CRC tissues and corresponding adjacent normal tissues from patients were collected from China-Japan Union Hospital of Jilin University. None of the patients who provided the sample had undergone preoperative chemotherapy or radiotherapy, and they all provided informed consent. The trial was approved by the Ethics Committee of Jilin University. All tissue samples were stored at −80 °C before use.

### 2.2. Database of Cancer Patients

Corresponding cancer stages and mRNA expression of ZEB2 and TWIST1 in colorectal patients were obtained by the online tool (https://cancergenome.nih.gov/) (accessed on 14 April 2022).

### 2.3. Antibodies and Reagents 

The following commercial antibodies and reagents were used: ZEB2 (ab138222, 1:500; Abcam, Cambridge, UK); TWIST1 (46702S, 1:500; Cell Signaling Technology, Danvers, MA, USA); E-cadherin (610181, 1:1000; BD Biosciences, San Diego, CA, USA); HDAC2 (5113T, 1:1000; Cell Signaling Technology); GAPDH (60004-1-Ig, 1:2000; Proteintech, Wuhan, China); ACTIN (66009-1-Ig, 1:2000; Proteintech); Tublin (11224-1-AP, 1:2000; Proteintech); GST (10000-0-Ig, 1:1000; Proteintech); HIS (66005-1-Ig, 1:1000; Proteintech); Flag (20543-1-AP, 1:1000; Proteintech); PRMT5 (18436-1-AP, 1:1000; Proteintech); RbAp48 (20364-1-AP, 1:1000; Proteintech); MTA2 (66195-1-Ig, 1:1000; Proteintech); Vimentin (10366-1-AP, 1:1000; Proteintech); Fibronectin (15613-1-AP, 1:1000; Proteintech); H3K56ac (39281, 5 μg/ChIP; Active Motif, Carlsbad, CA, USA); H4R3me2S (61187, 5 μg/ChIP; Active Motif). siRNAs and shRNAs were synthesized by GenePharma Co., Ltd. (Shanghai, China). The targeted sequences are listed in Supplementary Materials (Supplemental Table S1).

### 2.4. Cell Cultures and Stable Cell Lines

Human colorectal cancer cell line SW620, SW480, HT29, HCT116 were obtained from American Type Culture Collection (CCL-227, CCL-228, HTB-38, CCL-247). They were cultured according to the recommendations of the ATCC. 

Stable cell lines expressing ZEB2 was generated by transfection of pcDNA3.1-ZEB2-HA-IRES-EGFP, using Lipofectamine 2000 (Invitrogen, Camarillo, CA, USA). We established ZEB2, TWIST1 alone and co-silenced stable expression cell lines by transfection of shRNA plasmids. Stable monoclonal cells were collected using 400 µg/mL G418 screen.

### 2.5. Western Blotting and Immunoprecipitation

Standard methods of Western blotting and immunoprecipitation were used. Briefly, cells were lysed using RIPA buffer (Beyotime, Shanghai, China) and the supernatant was resolved by SDS-PAGE after centrifugation and transferred to PVDF membranes for Western blotting. Then, the membranes incubated with ECL luminescence reagent (Meilun Biotechnology Co., Ltd, Dalian, China) and exposed using Tanon 5200 Chemiluminescence Imaging System (Tanon Technology Co., Ltd, Shanghai, China). 

The glutathione S-transferase (GST) pull-down assays and immunoprecipitation experiments were performed as described before [21]. The supernatant was incubated FLAG-tagged beads (A2220; Sigma-Aldrich, St. Louis, MO, USA) or HA-tagged beads (11815016001; Roche, Mannheim, Germany) or Glutathione Beads (SA008005; Smart-Lifesciences Biotechnology Co., Ltd, Changzhou, China) for 4 h. The precipitates were then washed five times and analyzed by Western blotting.

### 2.6. Mass Spectrometry

Cell lysates were obtained from approximately 2 × 10^8^ HA-tagged ZEB2 stably overexpressing cells, which were co-incubated with equilibrated anti-HA affinity beads (Roche). After rotational binding at 4 °C for 2 h, C buffer (20 mM Tris-HCl, pH 8.0; 1.5 mM MgCl2; 0.42 M NaCl; 0.5 mM DTT; 0.2 mM EDTA) and rinse buffer (20 mM Tris-HCl, pH 8.0; 500 mM KCl; 1 mL EDTA; 1 mM DTT; 10% glycerol; 0.1% NP40) washed beads to remove heteroproteins. The beads were then washed twice with BC100 buffer (20 mM Tris-HCl, pH 8.0; 100 mM KCl; 1 mL EDTA; 1 mM DTT; 10% glycerol) to replace the high salt environment with a low salt environment. The beads were boiled in SDS loading buffer for 10 min. After centrifugation, supernatants were resolved on sodium dodecyl sulfate (SDS)-polyacrylamide gels, and subjected to liquid chromatographytandem mass spectrometry (LC-MS/MS) sequencing and data analysis.

### 2.7. Chromatin Immunoprecipitation Assay

Chromatin immunoprecipitation (ChIP) assays were performed as described [25]. Briefly, the cells were fixed in formaldehyde solution (Sigma-Aldrich, St. Louis, MO, USA) and sonicated on ice. Precleared lysates were incubated with antibodies overnight at 4 °C. Immunocomplexes were collected using the Chip Assay Kit (Millipore, Burlington, MA, USA) for qPCR analysis. The primers sequences are shown in Supplementary Materials (Supplemental Table S1).

### 2.8. Quantitative Real-Time PCR 

Total RNA was isolated using TRIzol (Invitrogen) and was synthesized complementary DNA using PrimeScript^™^ RT reagent Kit with gDNA Eraser (TaKaRa, Shiga, Japan) following the manufacturer’s protocol. Quantitative PCR was used TransStart^®^ Green qPCR SuperMix (TransGen, Beijing, China). Gene expression levels were quantified using the comparative Ct method. All data were normalized relative to β-actin as well as to the respective controls. Primer sequences are shown in Supplementary Materials (Supplemental Table S1).

### 2.9. Luciferase Assay

For luciferase assays, cells were transfected with E-cadherin-promoter plasmid containing luciferase concurrently with Renilla control. Luciferase assay was performed using Dual-Luciferase Reporter Assay system (E1910; Promega, Madison, WI, USA) as previously described [26].

### 2.10. Cell Migration and Invasion

Cell migration was assessed using wound-healing assays and transwell migration assay. Wound healing assays were performed as described previously [27,28]. Cell invasion was measured using BioCoat Matrigel invasion chambers (BD Biosciences, San Diego, CA, USA). Transwell migration assay and transwell invasion assays were performed as previously described [29]. SW480 cells (overexpressing ZEB2 or control) were analyzed by wound healing assay and transwell invasion assay. SW620 cells (SW620-sgNC, SW620-sgTWIST1, SW620-sgZEB2; SW620-shNC, SW620-shTWIST1, SW620-shZEB2 and SW620-shZEB2-shTWIST1) were analyzed by transwell migration experiment, wound healing assay and transwell invasion assay. HT29 cells (overexpressing ZEB2 or control) were analyzed by transwell migration experiment and transwell invasion assay. 

### 2.11. Liver Metastasis Model

Female BALB/c nude mice (4–5 weeks old, Charles River, Beijing, China) were used in this experiment. All animal studies were conducted in accordance with the Ethical Committee of Care and Use of Laboratory Animals at Jilin University and with institutional guidelines. Mice were injected into the tail veil with cells (2 × 10^6^ cells for shRNA, 8 mice/group). After 35 days, the mice were sacrificed. Liver metastatic nodules were examined macroscopically or detected in paraffin, sectioned, and stained with H&E. 

As for the survival assay, mice were injected into the tail veil with cells (2 × 10^6^ cells for shRNA, 10 mice/group). 

As for the treating assay, mice were injected with SW620 (2 × 10^6^ cells/mouse) injected into the tail veil (8 mice/group). After one week, Santacruzamate A (3 mg/kg, S7595; Selleck, Houston, Texas, USA) was given intravenously (i.v.) once every three days. GSK3326595 (100 mg/kg, twice daily, S8664; Selleck, Houston, Texas, USA) was given i.v. once every ten days. After 35 days, the mice were sacrificed. Then, nodules were paraffin-embedded, sectioned, and stained with H&E. 

### 2.12. Statistical Analysis

Statistical analysis was performed using Prism software (GraphPad Prism 9). The results are expressed as mean values ± SD. Significant differences were assessed using unpaired two-tailed unpaired *t*-tests, one-way ANOVA with Dunnett’s multiple comparisons test or two-way ANOVA with Sidak’s multiple comparisons test. Values of *p* below 0.05 were considered significant. * *p* < 0.05, ** *p* < 0.01, *** *p* < 0.001.

## 3. Results

### 3.1. ZEB2 and TWIST1 Are Significantly Upregulated during TGF-β-Induced EMT in CRC Cells and Patient Tissues

**Figure 1:**
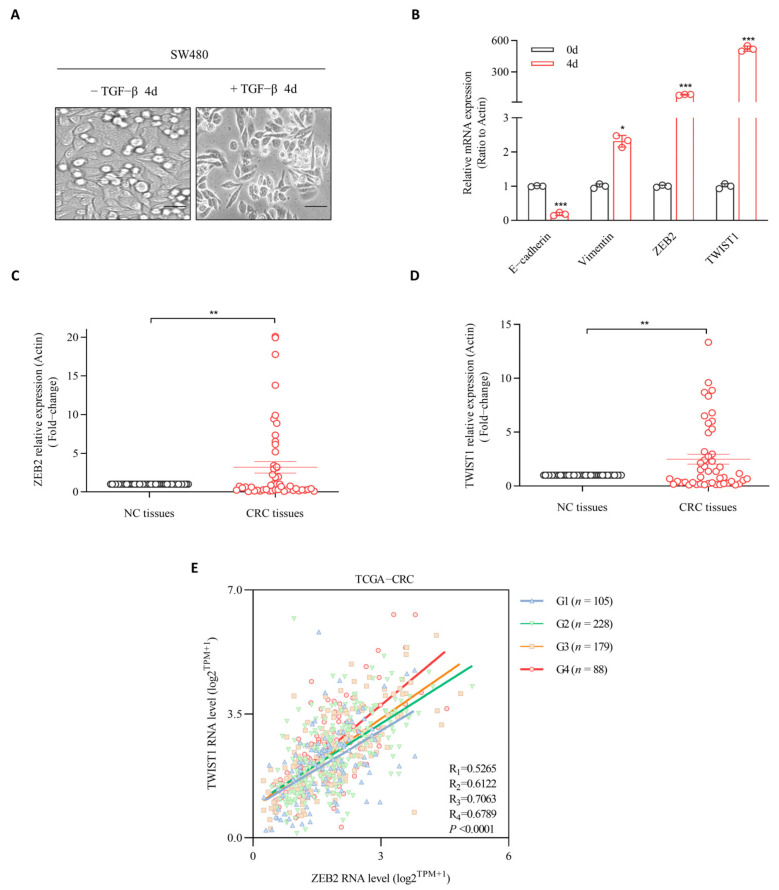
ZEB2 and TWIST1 are both remarkably upregulated during TGF-β-induced EMT in CRC cells and patient tissues. (**A**) Colon cancer cell line SW480 was treated with TGF-β1 (15 ng/mL) for 4 days; morphological changes were taken at ×10 magnification. Scale bars, 50 µm. (**B**) Relative mRNA levels analyses are shown as means ± SD. * *p* ≤ 0.05; *** *p* ≤ 0.001. (**C**,**D**) Expression levels of ZEB2 and TWIST1 in colon carcinoma in 49 paired CRC and NC tissues by qRT-PCR analysis. Results represent mean ± SD; ** *p* ≤ 0.01. (**E**) The positive correlation between expression of ZEB2 and TWIST1 target genes involved in various biological outcomes in CRC.

To determine the main transcriptional regulators during CRC metastasis, we quantified EMT transcriptional factors during the TGF-β-induced EMT process in the nonmetastatic CRC cell line SW480, by treating the cells with 15 ng/mL TGF-β1 for 4 days. Our results showed that the treatment induced SW480 cells to acquire mesenchymal phenotype (Figure 1A). Epithelial markers, including E-cadherin, were significantly downregulated, while mesenchymal markers, including vimentin, were upregulated (Figure 1B). In addition, we found that the expression levels of Snail1 and ZEB1 were either unchanged or slightly increased during EMT in SW480 cells (Supplementary Figure S1A). In contrast, the level of ZEB2 increased by almost 500 times after EMT induction in CRC cells. In parallel, the level of TWIST1 was also remarkably upregulated upon the downregulation of E-cadherin after TGF-β1 treatment (Figure 1B). To confirm the role of ZEB2 and TWIST1 in CRC tissues, we studied their expression levels. Our results showed that of ZEB2 expression level was markedly increased in CRC tissues. Similarly, the mRNA level of TWIST1 was two-fold higher in CRC tissues than in adjacent noncancerous tissues (Figure 1C,D). In contrast, the level of other EMT transcription factors, including Snail1 and ZEB1, remained unchanged in the CRC and control tissues (Supplementary Figure S1B,C). Furthermore, through the TCGA database we found that the mRNA expression levels of ZEB2 and TWIST1 were positively correlated across cancer malignancy stages (G1–G4) in 600 CRC patients (Figure 1E). The correlation between the mRNA levels of ZEB2 and TWIST1 was progressively stronger in colorectal cancers with moderate malignancy at G2 level and high malignancy at G3–G4 level, compared to those with low malignancy at G1 level. Therefore, our results suggest that ZEB2 and TWIST1 might be the master regulatory transcriptional factors involved in the progression of CRC. 

### 3.2. ZEB2 and TWIST1 Contribute to the EMT, Migration, and Invasion Processes in CRC

**Figure 2:**
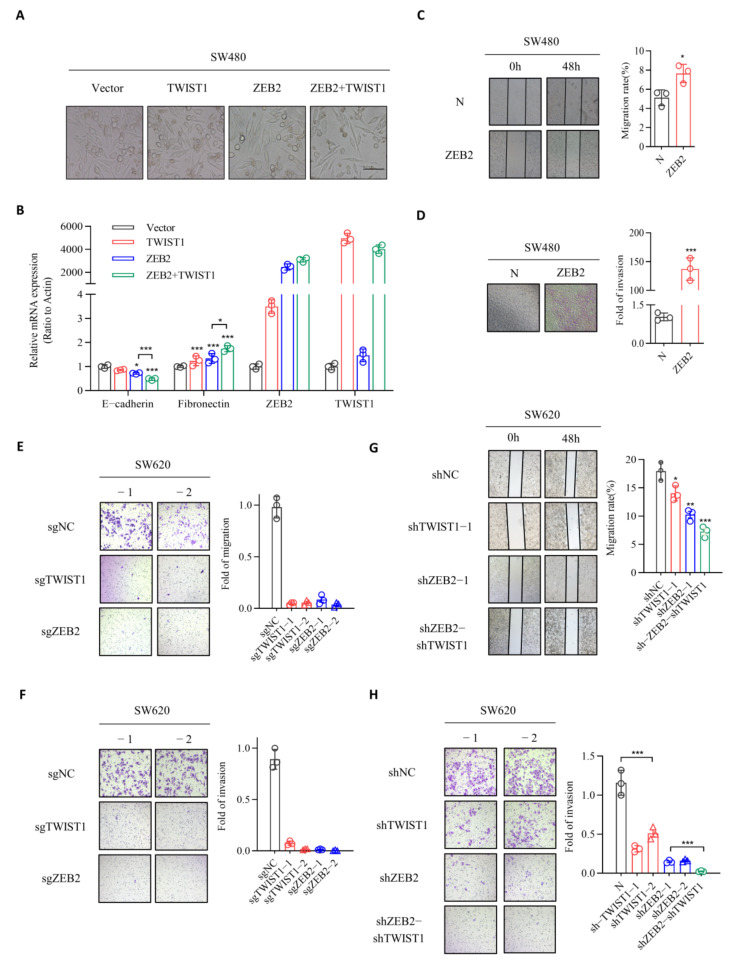
ZEB2 and TWIST1 both contribute to EMT, migration, and invasion processes of CRC. (**A**) The images of cell morphology about SW480+vector, SW480+TWIST1, SW480+ZEB2, and SW480+ZEB2+TWIST1 were captured by phase-contrast microscopy at ×20 magnification. Scale bars, 20 µm. (**B**) qRT-PCR analysis for mRNA expression levels of the EMT related genes E-cadherin, Fibronectin, ZEB2, TWIST1. (**C**,**D**) The migration and invasion abilities of SW480 cells (overexpressing ZEB2 or control) were analyzed by wound healing assays and transwell invasion assays. Data in the histogram are shown as the mean ± SD from three independent experiments. (**E**–**H**) The migration and invasion abilities of SW620 cells (SW620-sgNC, SW620-sgTWIST1, SW620-sgZEB2; SW620-shNC, SW620-shTWIST1, SW620-shZEB2, and SW620-shZEB2-shTWIST1) were analyzed by transwell migration experiment, wound healing assays, and transwell invasion assays. Data in the histogram are shown as the mean ± SD from three independent experiments. * *p* ≤ 0.05; ** *p* ≤ 0.01; *** *p* ≤ 0.001.

To validate the function of ZEB2 and TWIST1 during EMT in CRC cells, we overexpressed in SW480 cells. Cells overexpressing either ZEB2 or TWIST1, or both ZEB2 and TWIST1 were referred to as SW480+ZEB2, SW480+TWIST1 and SW480+ZEB2+TWIST1, respectively. As shown in Figure 2, all cells showed a fibroblastic, mesenchymal morphology, whereas the respective control cells showed an epithelial phenotype (Figure 2A). Overexpression of ZEB2 SW480 cells significantly reduced the expression of E-cadherin and increased the levels of fibronectin at both mRNA and protein levels (Figure 2B). Similarly, we observed a significant loss of E-cadherin and gain of mesenchymal markers including fibronectin in SW480+TWIST1 cells. These effects were significantly enhanced in SW480+ZEB2+TWIST1 cells. In addition, our results showed that the knockdown of either ZEB2 or TWIST1 in SW620 cells resulted in the increased expression of E-cadherin and decreased levels of mesenchymal markers (Supplementary Figure S2). These effects were markedly pronounced in SW620 cells following the combined knockdown of ZEB2 and TWIST1, proving that ZEB2 and TWIST1 are the key transcriptional regulators for E-cadherin repression in the EMT process in CRC cells. We then established ZEB2 over expression intestinal cancer stable transitional cell lines (Supplementary Figure S3). We successfully established ZEB2 and TWIST1 knockdown intestinal cancer stable transitional cell lines by sgRNA and shRNA (Supplementary Figure S4A,B). We investigated the roles of ZEB2 and TWIST1 in the migration-promoting potential of CRC cells using the wound healing assay. Compared to control cells, the overexpression of ZEB2 in non-metastatic CRC cell line SW480 markedly enhanced the migratory ability of cells (Figure 2C). Likewise, overexpression of ZEB2 in HT29 cells with low metastaticity significantly enhanced cell migration compared to controls (Supplementary Figure S5A). In contrast, the ZEB2 knockdown significantly reduced the migration rate of SW620 cells (Figure 2E,G). To verify the invasion-stimulating ability of TWIST1 and ZEB2, we performed transwell assay, which revealed that ZEB2 overexpression increased the invasive ability of SW480 cells and HT29 cells (Figure 2D, Supplementary Figure S5B), whereas knockdown in SW620 reduced it (Figure 2F,H). Similarly, these effects were also observed following TWIST1 knockdown in SW620 cells. Cumulatively, these data indicate that ZEB2 and TWIST1 play important roles in regulating EMT and promoting the migration and invasion of CRC.

### 3.3. ZEB2 Recruits TWIST1 to the Promoter of E-cadherin and Synergistically Represses the Transcription

**Figure 3:**
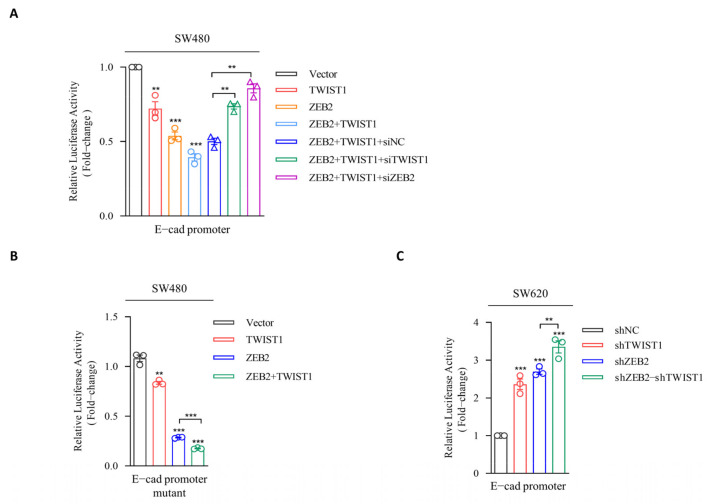
ZEB2 recruits TWIST1 to the E-cadherin promoter to synergistically repress transcription. (**A**) Luciferase reporter plasmid pGL3-E-cadherin promoter-Luc, Renilla (pRL-SV40), TWIST1, ZEB2, TWIST1+ZEB2, TWIST1+ZEB2+siNC, TWIST1+ZEB2+siTWSIT1, and TWIST1+ZEB2+siZEB2 were transfected into SW480 cells, 48 h later, luciferase activity was assayed and normalized to Renilla. (**B**) Plasmid pGL3-E-cadherin promoter mutant-Luc, Renilla, TWIST1, ZEB2, and TWIST1+ZEB2 were transfected into SW480 cells, 48 h later, luciferase activity was assayed and normalized to Renilla. (**C**) SW620 cells (SW620-shNC, SW620-shTWIST1-1, SW620-shZEB2-1, and SW620-shZEB2-shTWIST1) express plasmid pGL3-E-cadherin promoter-Luc. Luciferase activity was assayed and normalized to Renilla. Data points represent the mean ± SD (*n* = 3). ** *p* ≤ 0.01; *** *p* ≤ 0.001.

Next, we investigated the molecular mechanism of E-cadherin suppression by ZEB2 and TWIST1. We subcloned the truncated E-cadherin promoter region into the pGL3-basic luciferase vector to obtain pGL3-E-cadherin promoter-Luc vector, and evaluated the effects of ZEB2 and TWIST1 on E-cadherin transcription. Our results showed that luciferase activity was significantly decreased after co-transfection of pGL3-E-cadherin promoter-Luc and VR1012-ZEB2 in SW480 (Figure 3A). However, such inhibition was not observed in the case of E-box mutations in the E-cadherin promoter, suggesting that ZEB2 inhibits E-cadherin promoter by interacting with the E-box domain (Figure 3B). The overexpression of TWIST1 in SW480 cells could also significantly inhibit the luciferase activity. Surprisingly, ZEB2 overexpression remarkably enhanced the repressive activity of TWIST1 on the E-cadherin promoter. Reciprocally, overexpressed TWIST1 also had a positive effect on the transcriptional inhibition of E-cadherin by ZEB2 in SW480 cells (Figure 3A). Furthermore, ZEB2 downregulation abolished the TWIST1-induced E-cadherin suppression, whereas, TWIST1 downregulation had little effect the ZEB2-induced E-cadherin repression (Figure 3A). Similarly, the overexpression of both TWIST1 and ZEB2 alone in HT29 cells both significantly decreased the E-cadherin promoter activity, while the co-expression of both TWIST1 and ZEB2 enhanced the inhibition of E-cadherin promoter (Supplementary Figure S6). In addition, we knocked down the gene expression of ZEB2 and TWIST1 in SW620 cells and evaluated their influence on E-cadherin suppression (Figure 3C). Collectively, these results suggest that ZEB2 recruits TWIST1 to the E-cadherin promoter and then cooperatively inhibits the transcription of E-cadherin.

### 3.4. ZEB2 Interacts with TWIST1, PRMT5, and NuRD Complex to Form a Functional Multicomplex

**Figure 4:**
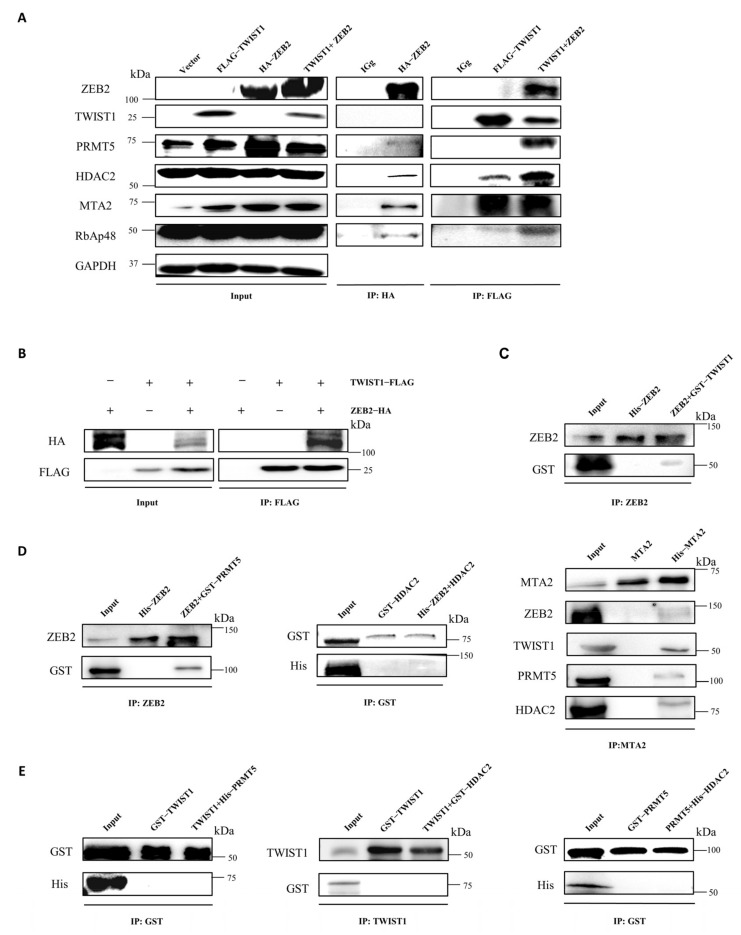
ZEB2 interacts with TWIST1, PRMT5, and NuRD complex to form a functional multicomplex. Purification of ZEB2-containing protein complexes in SW480 cells. Cellular extracts from SW480 cells expressing HA (control) or HA-ZEB2 were immunopurified with anti-HA affinity beads. Eluates were analyzed by SDS-PAGE and mass spectrometry. The detailed data are provided in Table 1. (**A**) Association of ZEB2 with TWIST1, PRMT5, and NuRD. Whole-cell lysates from SW480 cells (TWIST1, ZEB2, and TWIST1+ZEB2) were immunoprecipitated with antibodies and the precipitated proteins were blotted with antibodies against the indicated proteins. IgG served as a negative control. (**B**) Co-immunoprecipitation of FLAG-tagged TWIST1 and HA-tagged ZEB2 transiently transfected in SW480 cells. Extracts were immunoprecipitated with FLAG antibody, and bound ZEB2 was detected by Western blotting. (**C**–**E**) GST pull-down assays were performed by mixing GST-fused proteins and 6*His-fused proteins.

To better understand the molecular mechanisms underlying ZEB2-mediated repression of E-cadherin in CRC cells, we performed affinity purification and mass spectrometry by using ZEB2 overexpressed SW480 cells to identify the cofactors interacting with ZEB2. Mass spectrometric analysis revealed that ZEB2 formed a novel complex with MTA2 and RbAp48, which are the main subunits of NuRD complex. Notably, PRMT5, a histone arginine methyltransferase, is also part of the ZEB2 protein complex (Table 1). We further confirmed the ZEB2 novel complex by Western blotting using antibodies against the corresponding proteins, which revealed that in addition to the identified cofactors listed above, HDAC2, another core member of NuRD complex, was also detected in the immunoprecipitated products of ZEB2 in SW480 cells (Figure 4A). Also, PRMT5 and the subunits of NuRD complex, including HDAC2, MTA2 and RbAp48, were all detected in the immunoprecipitation products of ZEB2 in HT29, HCT116, and SW620 cells (Supplementary Figure S7). Moreover, we performed affinity purification in FLAG-TWIST1 overexpressed SW480 cells. TWIST1 also co-purified with NuRD complex, including MTA2, HDAC2, and RbAp48 in SW480 cells, which is consistent with previous findings in other cancer cell lines. However, unlike ZEB2, PRMT5 was not present in the TWIST1 protein complex (Figure 4A). We also failed to detect TWIST1 in the ZEB2-protein complex and vice versa, which might be due to the very low expression levels of ZEB2 and TWIST1 expression in SW480 cells (Supplementary Figure S8). Furthermore, we checked the components of ZEB2 or TWIST1 complexes in SW480 cells overexpressing both ZEB2 and TWIST1 (Figure 4B). Our results showed that in these cells, ZEB2 bound to TWIST1. Notably, ZEB2 bound with more NuRD complex when TWIST1 was overexpressed, suggesting that ZEB2 recruited more NuRD complexes to the E-cadherin promoter by interacting with TWIST1. In addition, we successfully detected PRMT5 in TWIST1/NuRD complex upon ZEB2 overexpression (Figure 4A). Therefore, our results indicate that ZEB2 interacts with TWIST1, PRMT5, and NuRD complex to form a functional multicomplex in CRC cells. 

To further verify the physical interactions between ZEB2, TWIST1, PRMT5, and NuRD complex in vitro, we purified different tagged proteins in E. coli and performed pull-down experiments in vitro. The results of GST pull-down assay revealed that ZEB2 directly interacted with TWIST1 (Figure 4C). The subsequent pull-down assay using GST/His-fused ZEB2, PRMT5, and subunits of the NuRD complex showed that ZEB2 associated directly with PRMT5 and MTA2 (Figure 4D). TWIST1 directly interacted with MTA2, but not with PRMT5 or HDAC2, which is consistent with the results of affinity purification in vivo (Figure 4D,E). We also could not detect the direct binding between PRMT5 and HDAC2, suggesting that ZEB2 and TWIST1 recruit other components of NuRD complex through MTA2 (Figure 4E).These data strongly support the presence of the ZEB2/TWIST1/PRMT5/NuRD complex in CRC cells.

### 3.5. PRMT5 and HDAC2 Are Responsible for the ZEB2-Mediated E-cadherin Repression in CRC

**Figure 5:**
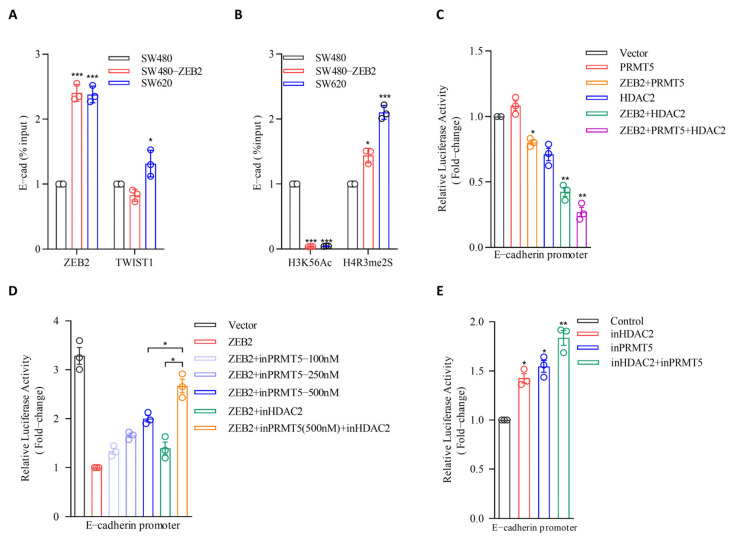
PRMT5 and HDAC2 are responsible for ZEB2-mediated E-cadherin repression in CRC. (**A**) ChIP assay of TWIST1 and ZEB2 levels at the E-cadherin promoter in the SW480-ZEB2 cell lines, their respective control cell lines, and SW620. ‘Percentage of input’ indicates the ratio of the DNA fragment of each promoter region bound by TWIST1 and ZEB2, to the total amount of input DNA without TWIST1 and ZEB2, specific antibody pull-down. (**B**) ChIP assay of H4R3 demethylation (H4R3me2S) and H3K56 acetylation (H3K56ac) levels at the E-cadherin promoter in SW480-ZEB2 cell lines, their respective control cell lines, and SW620. (**C**) SW480 cells express pGL3-E-cadherin promoter-Luc with plasmid ZEB2, PRMT5 or HDAC2, and luciferase activity was assayed, 48 h later, luciferase activity was assayed and normalized to Renilla. (**D**) SW480+Vector and SW480+ZEB2 cells express plasmid pGL3-E-cadherin promoter-Luc. InPRMT5 (GSK3326595) and inHDAC2 (Santacruzamate A) were added to the cell medium, and luciferase activity was assayed, 48 h later, luciferase activity was assayed and normalized to Renilla. (**E**) SW620 cells express plasmid pGL3-E-cadherin promoter-Luc. InPRMT5 (GSK3326595) and inHDAC2 (Santacruzamate A) were added to the medium, and luciferase activity was assayed, 48 h later, luciferase activity was assayed and normalized to Renilla. All the data are representative of three independent experiments. Data points represent the mean ± SD. * *p* ≤ 0.05; ** *p* ≤ 0.01; *** *p* ≤ 0.001.

To explore the function of the ZEB2/TWIST1/PRMT5/NuRD complex in regulating E-cadherin during EMT in CRC, we examined the levels of ZEB2 and TWIST1 on the E-cadherin promoter using ChIP assays. Our results showed that ZEB2 and TWIST1 levels were significantly enriched on the E-cadherin promoter in SW480-ZEB2 cells (Figure 5A). PRMT5 is a symmetrical H4R3me2 methyltransferase and HDAC2 is a H3K56 histone deacetylase. To determine whether the ZEB2/TWIST1/PRMT5/NuRD complex can epigenetically regulate E-cadherin transcription in CRC, we further examined the levels of histone modifications, including H4R3me2 and H3K56ac, on the E-cadherin promoter by ChIP assay. As shown in Figure 5, the H4R3me2 was upregulated in the E-cadherin promoter in SW480-ZEB2 cells, whereas H3K56ac was reduced (Figure 5B), indicating that the ZEB2 multicomplex mediates the transcriptional repression of the E-cadherin promoter through H4R3 methylation and H3K56 deacetylation.

Next, we investigated the involvement of PRMT5 and HDAC2 in ZEB2 and TWIST1-mediated E-cadherin suppression using luciferase reporter assay, which revealed that PRMT5 overexpression reduced luciferase activity in SW480-ZEB2 cells. However, when PRMT5 was overexpressed alone in SW480 cells, we could not detect a decrease in luciferase activity, suggesting that ZEB2 mediates the association between PRMT5 and the E-cadherin promoter (Figure 5C). We also found that HDAC2 repressed the activity of the E-cadherin promoter and this repressive function is partial ZEB2 dependent (Figure 5C). 

Furthermore, to confirm the repressive function of PRMT5 and HDAC2 on the E-cadherin promoter, we treated SW480-ZEB2 cells with the PRMT5 inhibitor GSK3326595 and the HDAC2 inhibitor Santacruzamate A. As expected, both GSK3326595 and Santacruzamate A treatments rescued the ZEB2-mediated E-cadherin promoter suppression in SW480-ZEB2 cells, confirming that the ZEB2-mediated repression of E-cadherin in CRC cells is facilitated by PRMT5 and HDAC2. Notably, the simultaneous inhibition of PRMT5 and HDAC2 enhanced these observed effects, indicating that the PRMT5 and NuRD complex may act in a collaborative manner to simultaneously methylate H4R3 and deacetylate H3K56 histones (Figure 5D). We also verified the function of PRMT5 and HDAC2 on E-cadherin repression in SW620 cells. The inhibition of PRMT5 or HDACs alone in SW620 cells slightly upregulated the activity of the E-cadherin promoter, whereas inhibition of both proteins remarkably rescued the ZEB2-mediated E-cadherin promoter repression (Figure 5E). Collectively, these results indicate that ZEB2 cooperates with TWIST1 to repress the transcription of E-cadherin by recruiting PRMT5 and NuRD complex, as well as mediating H4R3 methylation and H3K56 deacetylation on the promoter region.

### 3.6. PRMT5 and HDAC2 Mediate the Migration-, Invasion-, and Metastasis-Promoting Properties of ZEB2

**Figure 6:**
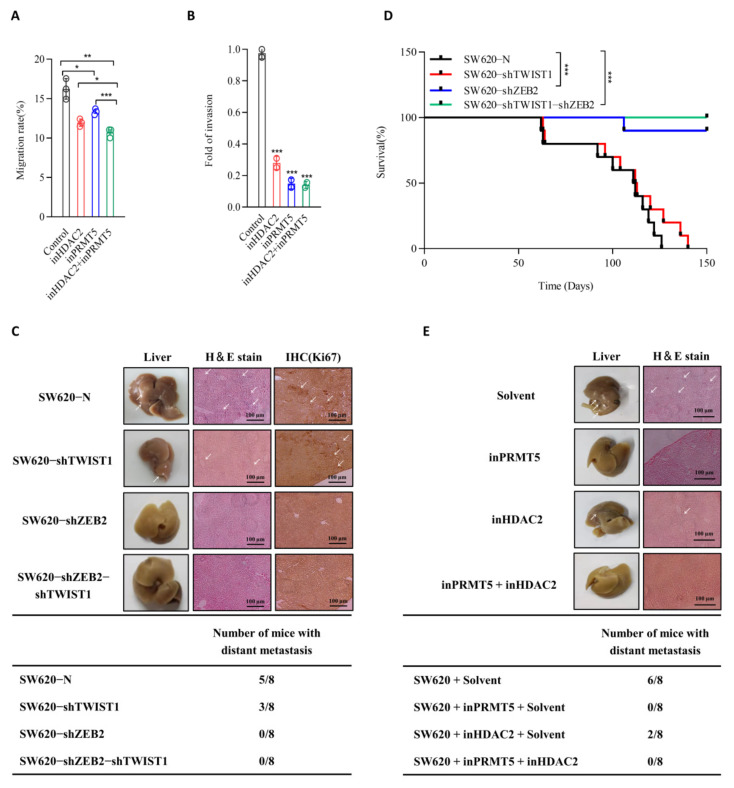
PRMT5 and HDAC2 are responsible for the migration, invasion, and metastasis triggered by ZEB2 in CRC cells. (**A**,**B**) Analysis of wound healing assays of the migratory ability and transwell invasion assays of the invasiveness of SW620 cells treated with GSK3326595 (PRMT5 inhibitor) and Santacruzamate A (HDAC2 inhibitor). Statistical analysis of migration and invasion are shown in the bar graph (mean ± SD from three independent experiments). (**C**) Total number of mice with distant metastasis 40 days after injection of SW620-shZEB2, SW620-shTWIST1, SW620-shZEB2-shTWIST1, and SW620 control cells. SW620-shZEB2, SW620-shTWIST1, SW620-shZEB2-shTWIST1 and respective SW620 control cells were injected into nude mice. After 40 days, the mice were sacrificed and their livers were dissected. Liver metastatic nodules were examined macroscopically, and paraffin-embedded sections were subjected to H&E and Ki67 staining. Arrowheads indicate liver metastases. Phase contrast images were taken at ×4 magnification. (**D**) A total of 2 × 10^6^ SW620 were injected into the tail veil of nude mice. Metastasis was suppressed by GSK3326595 or Santacruzamate A treatment 35 days after injection. The survival time of the mice was analyzed by Kaplan–Meier survival analysis. (**E**) Total number of mice with distant metastasis 40 days after injection of SW620 cells with GSK3326595 and Santacruzamate A. SW620 cells, GSK3326595 and Santacruzamate A treatments were injected into nude mice. After 40 days, the mice were sacrificed and their livers were dissected. Liver metastatic nodules were examined macroscopically, and paraffin-embedded sections were subjected to H&E staining. * *p* ≤ 0.05; ** *p* ≤ 0.01; *** *p* ≤ 0.001.

Given that PRMT5 and HDAC2 are responsible for the ZEB2-mediated repression of E-cadherin in CRC cells, we next investigated their cooperative functions in the invasion and metastasis of CRC. Since the downregulation of ZEB2 or TWIST1 in SW620 cells reduced the cell migratory capacity (Figure 2E), we treated these cell lines with GSK3326595 and Santacruzamate A and compared their migration ability to that of untreated cells. Our results revealed that PRMT5 inhibition significantly reduced the migratory ability of SW620 cells (Figure 6A). The functional suppression of PRMT5 and HDAC activity also decreased the invasive potential of the SW620 cells (Figure 6B). At the same time, we also used sgRNA to knock down the gene expression levels of PRMT5, HDAC2 and MTA2, and the results showed a similar trend (Supplementary Figure S9). These results indicated that PRMT5 and HDAC2 cooperate with ZEB2 and TWIST1 to enhance the migration and invasion of CRC cells in vitro.

Next, we generated an in vivo xenograft model of metastasis, in which CRC cells were directly injected into the tail vein of nude mice to generate hepatic metastases. SW620-shZEB2, SW620-shTWIST1, SW620-shZEB2-shTWIST1, and SW620 control cells were injected into nude mice to evaluate their metastatic potential. Compared with SW620-N-injected mice, mice injected with SW620-shTWIST1 cells had reduced liver metastasis. Notably, ZEB2 downregulation in SW620 completely abolished the metastatic potential of CRC cells in mice. The double knockdown of ZEB2 and TWIST1 in SW620 cells also abolished the liver metastasis, indicating that ZEB2 and TWIST1 promote invasion and metastasis of CRC in vivo (Figure 6C). To further explore the role of ZEB2 and TWIST1 in CRC invasion and metastasis, we examined the survival time of mice in which were injected into SW620-N, SW620-shZEB2, SW620-shTWIST1, SW620-shZEB2-shTWIST1. The knockdown of TWIST1 in SW620 cells virtually no effect to survival time in mice (injected with cells), the knockdown of ZEB2 and double knockdown of ZEB2 and TWIST1 both successfully increased survival time, compared to that observed in control mice injected into SW620-N (Figure 6D). 

**Figure 7:**
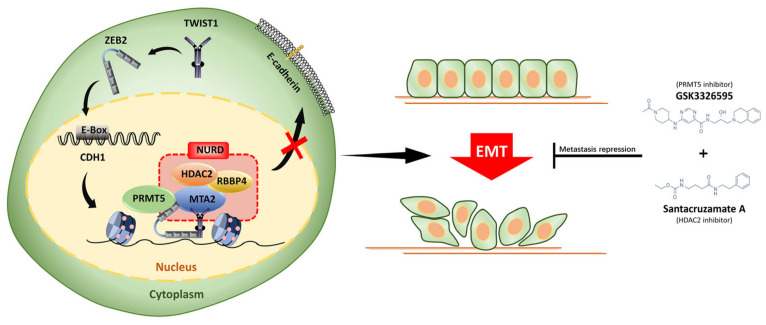
A proposed working model of the role of the ZEB2/TWIST1/PRMT5/NuRD complex in EMT and tumor metastasis in CRC.

To further explore the role of PRMT5 and HDAC2 in CRC invasion and metastasis, we treated SW620 cells with inPRMT5 and inHDAC2. Both treatments reduced liver metastasis of CRC cells and completely inhibited the distant metastasis of SW620 cells in nude mice (Figure 6E). Altogether, our data provide compelling evidence that ZEB2 interacts with TWIST1 to recruit PRMT5 and NuRD complex to form a novel complex and epigenetically suppresses E-cadherin transcription, thereby inducing the EMT process and metastasis in CRC (Figure 7). 

## 4. Discussion

Several EMT transcription factors are involved in the metastasis of CRC. ZEB2 was shown to promote tumor invasion and metastasis and is associated with poor prognosis in CRC patients [10,30]. Several studies identified Snail2 as a transcriptional repressor of E-cadherin, and a predictive factor for metastasis in CRC patients [7,31]. Most CRC samples have a high expression level of TWIST1, greater than that of Snail1 and Snail2 [9,31,32]. Nevertheless, these studies only predicted the relationship between transcriptional factors and CRC metastasis by analyzing data from patient tissues, and few studies have investigated EMT transcription factors in CRC both in vitro and in vivo. Here, we show that during TGF-β-induced EMT process in CRC cells, only ZEB2 and TWIST1 were strikingly upregulated. They were also significantly enriched in tissues of patients with CRC. The co-expression of ZEB2 and TWIST1 has been previously reported in patients with oral squamous cell carcinoma [11]. However, it remains unclear whether there is a direct link between ZEB2 and TWIST1 in oral squamous cell carcinoma and other carcinomas. Our results revealed a specific interaction between ZEB2 and TWIST1 in CRC, and confirmed that ZEB2 and TWIST1 are the key drivers of EMT in CRC. Furthermore, we demonstrated that ZEB2 could recruit TWIST1 to E-cadherin promoter and synergistically inhibit its transcription in CRC cells. Hence, the ZEB2/TWIST1 co-repression of E-cadherin appears to be ubiquitous and crucial, providing potential targets for clinical diagnosis and therapeutical design for malignant carcinomas. 

In addition, EMT is epigenetically regulated by a series of histone modification enzymes in several carcinomas. PRMT5, a histone arginine methyltransferase, is highly expressed in a wide range of malignant carcinomas, including ovarian, lung, and gastric cancers [33,34,35]. However, the specific role of PRMT5 in the EMT and metastasis of CRC remains unknown. We found that in CRC, ZEB2 directly associates with PRMT5, recruits it to the promoter region of E-cadherin, catalyzes the repressive H4R3me2 modification, and finally triggers the transcriptional repression of E-cadherin. Furthermore, PRMT5 inhibitor inhibited liver metastasis in CRC cells in a mouse model. However, although TWIST1 can also inhibit the expression of E-cadherin in CRC cells, TWIST1 did not interact with PRMT5, suggesting a complicated but hierarchical regulatory network of EMT in CRC. 

Recently, increasing studies have shown that HDAC2 plays a crucial role in the carcinogenesis of CRC. Benard et al. found an increase in HDAC2 and a decrease in H3K56 acetylation in CRC tissues [36]. Qi and colleagues also reported that HDAC2 promotes EMT and metastasis in CRC cells by binding to a novel lncRNA [37]. HDACs are usually part of a multimeric co-repressive complex which is recruited to the E-cadherin promoter. The NuRD complex is a major chromatin remodeling complex found in cells, and comprises the histone deacetylases HDAC1 and HDAC2 [38]. Recently, the NuRD complex has been shown to be implicated in the transcriptional repression of E-cadherin in tumor metastasis. Fu et al. found that TWIST1 directly interacts with the NuRD complex and cooperatively promotes EMT and metastasis in cancer cells [24]. Snail1 also forms a repressive complex with NuRD and PRMT5 to contribute to histone deacetylation and methylation of E-cadherin in cervical cancer [21]. Additionally, Manshouri and colleagues demonstrated that the ZEB1/NuRD complex inhibits TBC1D2b to stimulate E-cadherin internalization and promotes metastasis in lung cancer [39]. Nevertheless, the functions and regulatory mechanisms of the NuRD complex during the EMT and metastasis of CRC have not yet been defined. We revealed that, as ZEB2 and TWIST1 directly interact with the NuRD complex. Similar to the TWIST1/NuRD complex, the ZEB2/NuRD complex is also maintained by multiple interactions between different components. Within the complex ZEB2 was able to recruit more NuRD complexes, through TWIST1, to the E-cadherin promoter and remove more acetyl residues from H3K56, leading to the extreme repression of E-cadherin repression. Moreover, similar to Snail1 in cervical cancer, ZEB2 also binds to PRMT5 and the NuRD complex to form a multimeric transcriptionally repressive unit. Therefore, targeting these histone modification enzymes is a potential therapeutic strategy for malignant carcinomas. Indeed, in cell culture, inhibition of PRMT5 or HDAC2 downregulated the migratory and invasive potential of CRC cells. In a mouse model, treatment with a PRMT5 or HDAC2 inhibitor also prevented the distant metastasis of CRC cells. GSK3326595, a potent and selective PRMT5 inhibitor with demonstrated efficacy in multiple tumor models, was recently investigated in a phase II clinical study in human subjects with acute myeloid leukemia. A phase I study was also conducted to assess the safety and efficacy of GSK3326595 in adults with diverse solid tumors. Thus, our study has provided new insights into its potential role in the treatment of CRC in human subjects.

## 5. Conclusions

Overall, we revealed that ZEB2 could recruit TWIST1, PRMT5, and NuRD to form a repressive multicomplex and epigenetically suppresses the transcription of E-cadherin, thereby inducing the EMT process and metastasis in CRC. Our results also confirmed the therapeutic potential of epigenetic inhibitors in the treatment of CRC.

## Figures and Tables

**Table 1:** Identification and LC–MS based label free quantification of the binding partners of ZEB2 following HA affinity enrichment.

Accession ^a^	Peptide Count ^b^	Confidence Score ^c^	*p*-Valued ^d^	*q*-Valued ^d^	Protein Description
A0JP08	43 (43)	1595.43	<0.0001	<0.0001	Zinc finger E-box-binding homeobox 2
B4DV00	5 (5)	119.52	<0.0001	<0.0001	Protein arginine N-methyltransferase 5
Q09028	2 (2)	57.93	0.00073	0.00169	Histone-binding protein RBBP4
O94776	1 (1)	54.81	0.00040	0.00025	Metastasis-associated protein MTA2

^a^ Accession = FASTA database Protein ID. ^b^ Peptide count = the number of detected peptides (the number of unique peptides) used for quantification. ^c^ The protein confidence score was generated using Mascot. ^d^ The *p*-value, *q*-value and fold change were generated by Progenesis QI for proteomics.

## Data Availability

The datasets used and analyzed during the current study are available from the corresponding author on reasonable request.

## Supplementary Materials

The following supporting information can be downloaded at: https://www.mdpi.com/article/10.3390/cancers14143426/s1, Table S1: siRNAs sequences, sgRNAs and qPCR primer sequences; Figure S1: The expression levels of Snail1 and ZEB1 during TGF-β-induced EMT in CRC cells and patient tissues; Figure S2: The mRNA expression levels of the EMT-related genes with knockdown of ZEB2 or TWIST1 in CRC cells; Figure S3: The protein expression levels of the EMT-related genes with overexpression of ZEB2 in CRC cells; Figure S4: Establishment of ZEB2 and TWIST1 stably silenced colorectal cancer cell lines; Figure S5: Evaluation of tumor migration and invasion ability of overexpressing ZEB2 CRC cell lines; Figure S6: ZEB2 recruits TWIST1 to the E-cadherin promoter to synergistically repress transcription; Figure S7: ZEB2 interacts with TWIST1, PRMT5, and NuRD complex to form a functional multicomplex in different colorectal cancer cell lines; Figure S8: Expression of ZEB2, TWIST1 and E-cadherin in SW620 and SW480 cell lines; Figure S9: Evaluation of tumor migration and invasion ability of ZEB2 and TWIST1 stable knockout CRC cell lines; Figure S10: Original Western blot.

## Abbreviations

CRC	colorectal carcinoma
EMT	epithelial to mesenchymal transition
NuRD	nucleosome remodeling and deacetylase
PRMT5	Protein arginine methyltransferase 5
HDAC	Histone deacetylases
Co-IP	co-immunoprecipitation
inHDAC2	Santacruzamate A
inPRMT5	GSK3326595
