# Supplementary material for: ZEB2/TWIST1/PRMT5/NuRD Multicomplex Contributes to the Epigenetic Regulation of EMT and Metastasis in Colorectal Carcinoma
Source: Cancers (Basel). 2022 Jul 14;14(14):3426. doi: 10.3390/cancers14143426 (PMC9324012; doi:10.3390/cancers14143426)

ZEB2/TWIST1/PRMT5/NuRD multicomplex contributes to the epigenetic regulation of EMT and metastasis in colorectal carcinoma

Table S1. siRNAs sequences, sgRNAs and qPCR primer sequences.

**siRNA**	**Sequences (5′ – 3′)**
siZEB2 (1503)	Forward: 5′−GCACAUCAGCAGCAAGAAATT−3′
	Reverse: 5′−UUUCUUGCUGCUGAUGUGCTT−3′
siTWIST1(1577)	Forward: 5′−GGUGUCUAAAUGCAUUCAUTT−3′
	Reverse: 5′−AUGAAUGCAUUUAGACACCTT−3′
sgRNA	sequences for CRISPR knock out (5′ – 3′)
HDAC2 −1	TTCTGGTTTGTCATGTTTGA
HDAC2 −2	TCCAACATCGAGCAACATTA
PRMT5 −1	CCCTTCTCCGTCCCCGAGTT
PRMT5 −2	AGTTCATAGGCATTAGGTG
MTA2 −1	GATCCCAGATCGCCTAGTAG
MTA2 −2	AGGAACATCTTGGACCTCCG
ZEB2 −1	GAATCTCGTTGTTGTGCCAG
ZEB2 −2	ACTTGCGATTACCTGCTCCT
TWIST1 −1	CGGGAGTCCGCAGTCTTACG
TWIST1 −2	CTGTCGTCGGCCGGCGAGAC
Nontargeting contral	CACCGCTGAAAAAGGAAGGAGTTGA
Gene	Primer sequence
β −actin	Forward: 5′−CGTGGACATCCGCAAAGACC−3′
	Reverse: 5′−GGACTCGTCATACTCCTGCTTGC−3′
TWIST1	Forward: 5′−GGAGTCCGCAGTCTTACGAG−3′
	Reverse: 5′−TCTGGAGGACCTGGTAGAGG−3′
ZEB2	Forward: 5′−AACAACGAGATTCTACAAGCCTC−3′
	Reverse: 5′−TCGCGTTCCTCCAGTTTTCTT −3′
E −cadherin	Forward: 5′−TGCCCAGAAAATGAAAAAGG−3′
	Reverse: 5′−GTGTATGTGGCAATGCGTTC−3′
Fibronectin	Forward: 5′−CAGTGGGAGACCTCGAGAAG−3′
	Reverse: 5′−TCCCTCGGAACATCAGAAACC−3′
Vimentin	Forward: 5′−GAGAACTTTGCCGTTGAAGC−3′
	Reverse: 5′−GCTTCCTGTAGGTGGCAATC−3′
Snail1	Forward: 5′−TATGCTGCCTTCCCAGGCTTG−3′
	Reverse: 5′−ATGTGCATCTTGAGGGCACCC−3′
ZEB1	Forward: 5′−TTTAAGAGATAAGAAGCAACCG−3′
	Reverse: 5′−GACGGCGAGGACACGCGGCGA−3′
E −cadherin promoter	Forward: 5′−GCCCTTTCTGATCCCAGGTC−3′
	Reverse: 5′−TAGCCTGGAGTTGCTAGGGT−3′

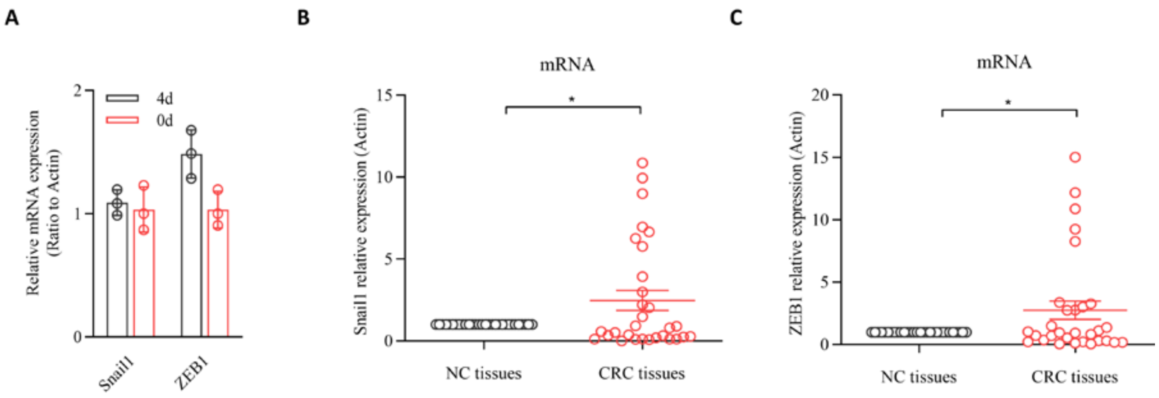

Figure S1. The expression levels of Snail1 and ZEB1 during TGF−β−induced EMT in CRC cells and patient tissues. **(A)** SW480 cells were treated with TGF−β1 (15 ng/ml) for 4 days; relative mRNA levels analyses are shown as means ± SD. **p* ≤ 0.05; ****p* ≤ 0.001. **(B)** Expression levels of Snail1 in colon carcinoma qRT−PCR analysis of Snail1 expression levels in 30 paired CRC and NC tissues. Data represent mean ± SD; **p* ≤ 0.05, ***p* ≤ 0.01. **(C)** Expression levels of ZEB1 in colon carcinoma qRT−PCR analysis of the expression levels of ZEB1 in 30 paired CRC and NC tissues. Data represent mean ± SD; **p* ≤ 0.05, ***p* ≤ 0.01.

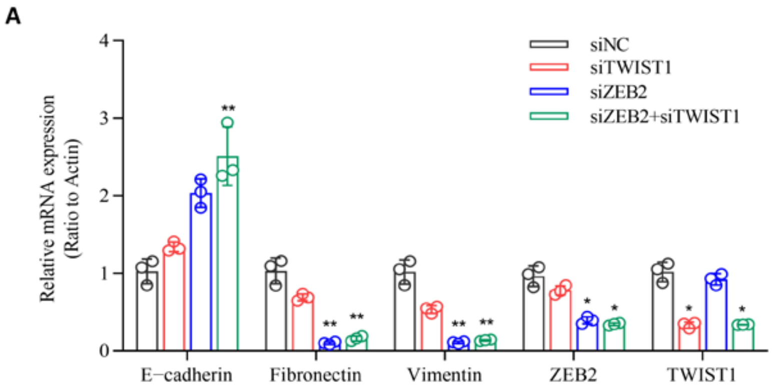

Figure S2. The mRNA expression levels of the EMT−related genes with knockdown of ZEB2 or TWIST1 in CRC cells. **(A)** qRT−PCR analysis for mRNA expression levels of the EMT−related genes E−cadherin, Fibronectin, Vimentin, ZEB2, TWIST1. Data represent mean ± SD; **p* ≤ 0.05, ***p* ≤ 0.01.

A

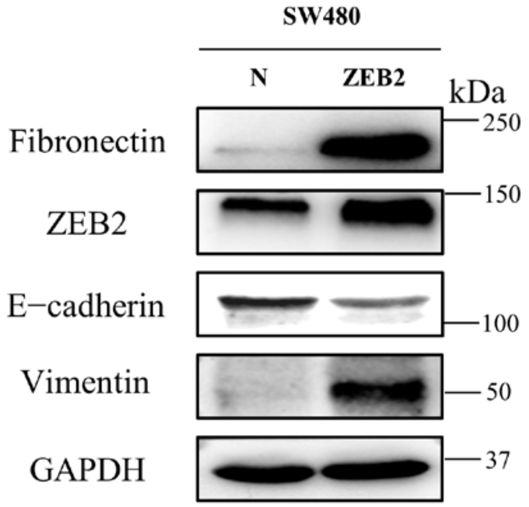

Figure S3. The protein expression levels of the EMT−related genes with overexpression of ZEB2 in CRC cells. **(A)** Expression of E−cadherin, fibronectin, vimentin, and ZEB2 in these cells (SW480−N, SW480−ZEB2) was analyzed by Western blotting.

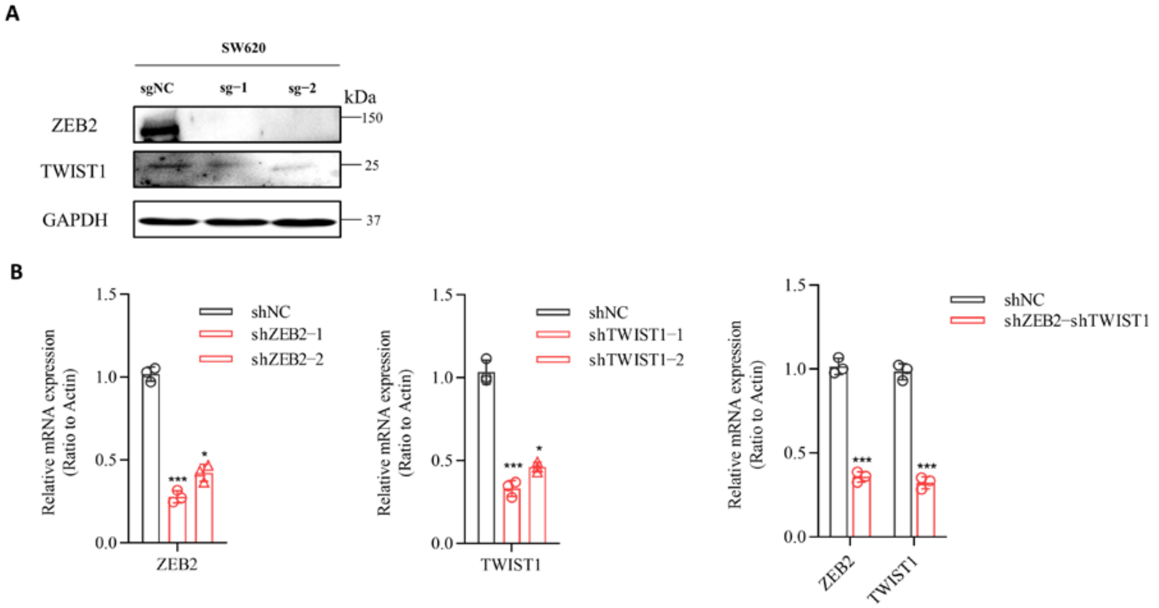

Figure S4. Establishment of ZEB2 and TWIST1 stably silenced colorectal cancer cell lines. (**A**) Expression of ZEB2 and TWIST1 in SW620−sgNC, SW620−sgZEB2−1, SW620−sgZEB2−2, SW620−sgTWIST1−1, SW620−sgTWIST1−2 cells were analyzed by Western blotting. **(B)** Expression of ZEB2 or TWIST1 in SW620−shNC, SW620−shZEB2−1, SW620−shZEB2−2, SW620−shTWIST1−1, SW620−shTWIST1−2 and SW620−shZEB2−shTWIST1 cells were analyzed by qPCR and shown as "relative mRNA levels." Data represent the mean ± SD. *$p \leq 0.05$; **$p \leq 0.01$; ***$p \leq 0.001$.

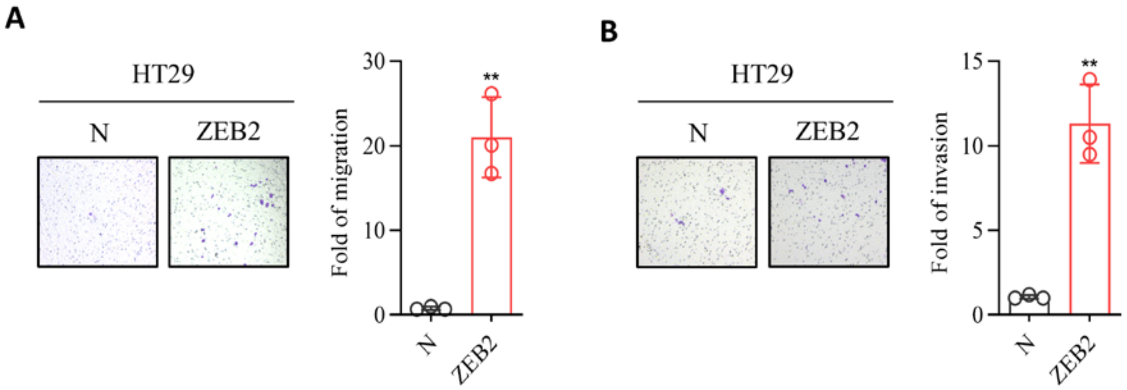

Figure S5. Evaluation of tumor migration and invasion ability of overexpressing ZEB2 CRC cell lines. **(A−B)** The migration and invasion abilities of HT29 cells (overexpressing ZEB2 or control) were analyzed by transwell migration experiment and transwell invasion assays. Data in the histogram are shown as the mean ± SD from three independent experiments. $^{**}p \leq 0.01$.

A

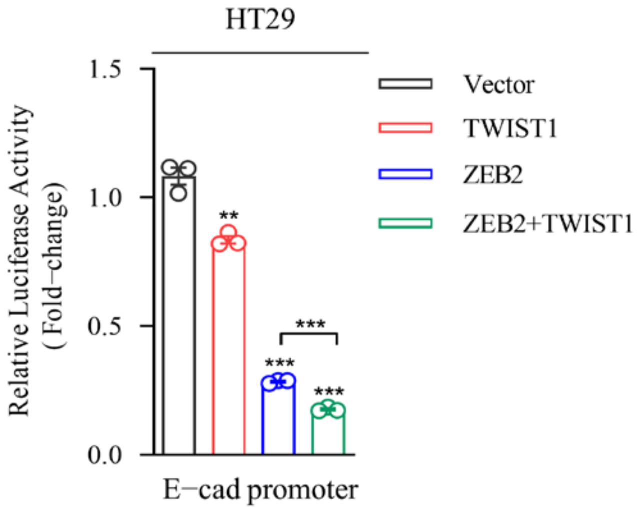

Figure S6. ZEB2 recruits TWIST1 to the E−cadherin promoter to synergistically repress transcription. **(A)** Luciferase reporter plasmid pGL3−E−cadherin promoter−Luc, Renilla (pRL−SV40), TWIST1, ZEB2 and TWIST1+ ZEB2 were transfected into HT29 cells, 48 h later, luciferase activity was assayed and normalized to Renilla. Data points represent the mean ± SD (n = 3). ** $p \le 0.01$; *** $p \le 0.001$.

A

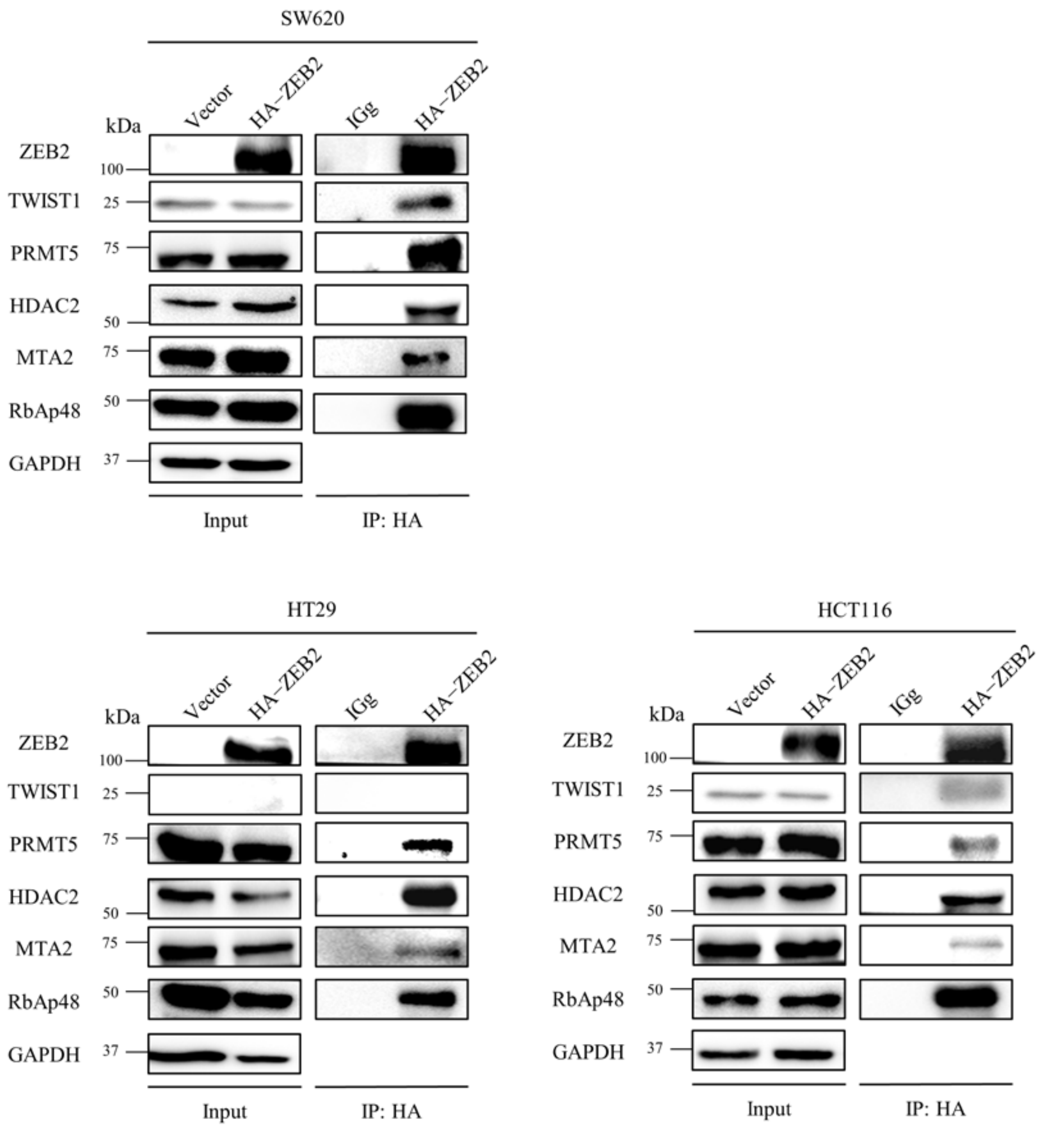

Figure S7. ZEB2 interacts with TWIST1, PRMT5, and NuRD complex to form a functional multi-complex in different colorectal cancer cell lines. **(A)** Purification of ZEB2−containing protein complexes in SW620, HT29 and HCT116 cells. Cellular extracts from these cells which were transfected with vector (control) or plasmid HA−ZEB2 were immunopurified with anti−HA affinity beads with antibodies and the precipitated proteins were blotted with antibodies against the indicated proteins. IgG served as a negative control.

A

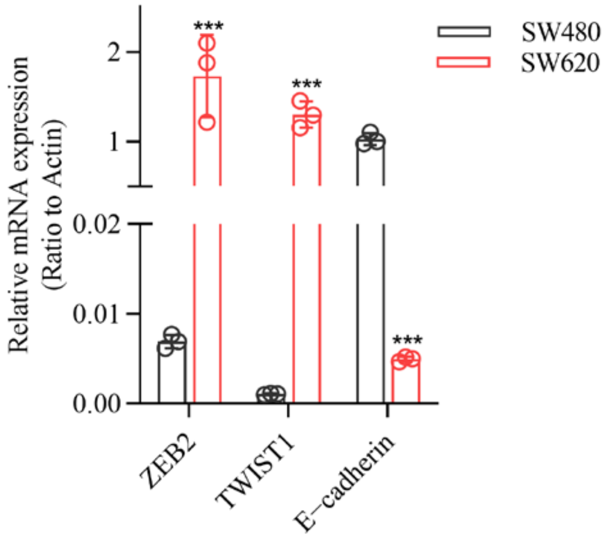

Figure S8. Expression of ZEB2, TWIST1 and E−cadherin in SW620 and SW480 cell lines. **(A)** Expression of ZEB2, TWIST1 and E−cadherin in SW620 and SW480 cell lines was analyzed by qPCR and shown as “relative mRNA levels.” Data represent the mean ± SD. *$p \leq 0.05$; **$p \leq 0.01$; ***$p \leq 0.001$.

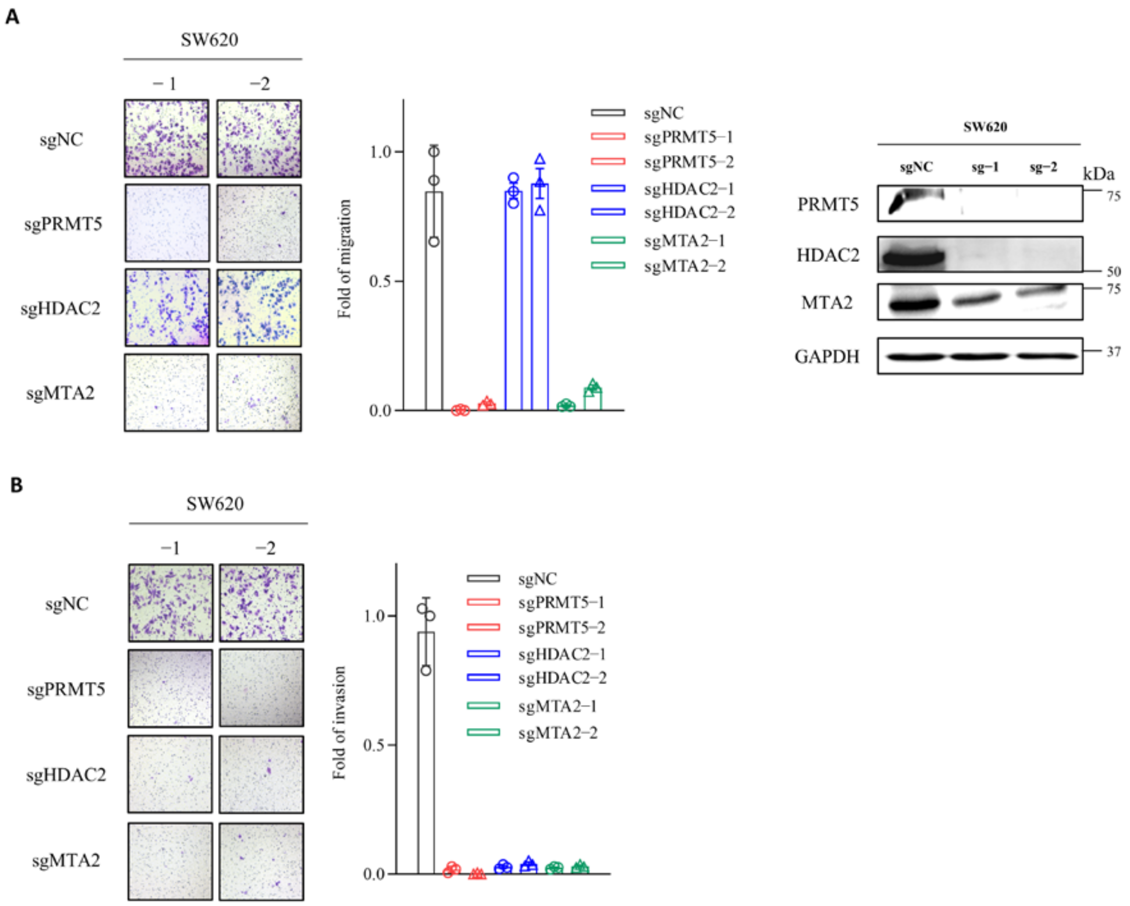

Figure S9. Evaluation of tumor migration and invasion ability of ZEB2 and TWIST1 stable knock-out CRC cell lines. **(A–B)** The migration abilities and invasion abilities of SW620 cells (SW620−sgNC, SW620−sgPRMT5, SW620−sgMTA2 and SW620−sgHDAC2) were analyzed by transwell migration experiment and transwell invasion assays. The statistical analysis is shown in the bar graph (mean ± SD from three independent experiments), and a representative experiment is shown in the right panel.

Figure S10 Original Western blot**.**

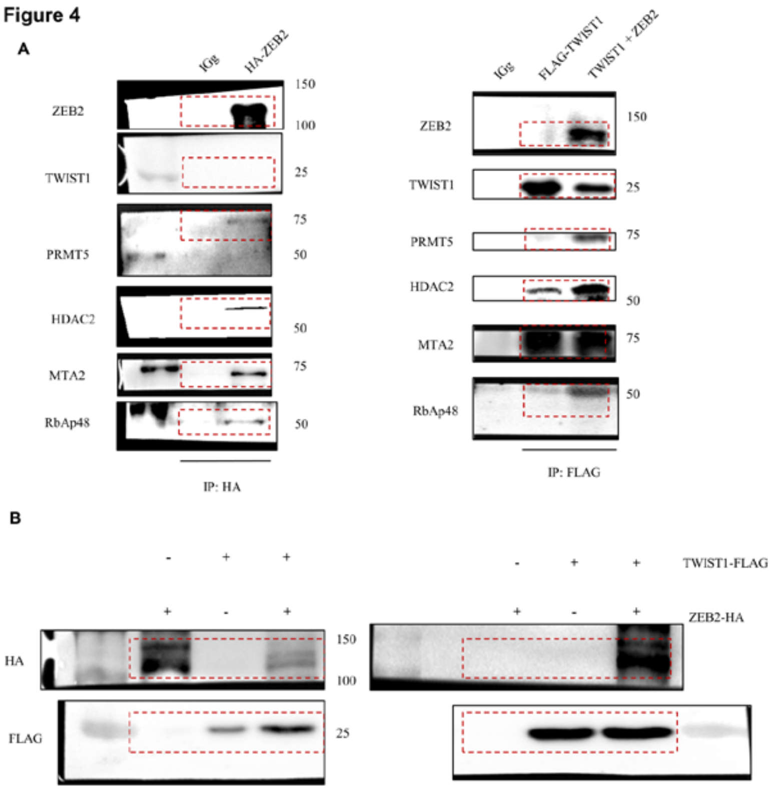

Figure 4

A

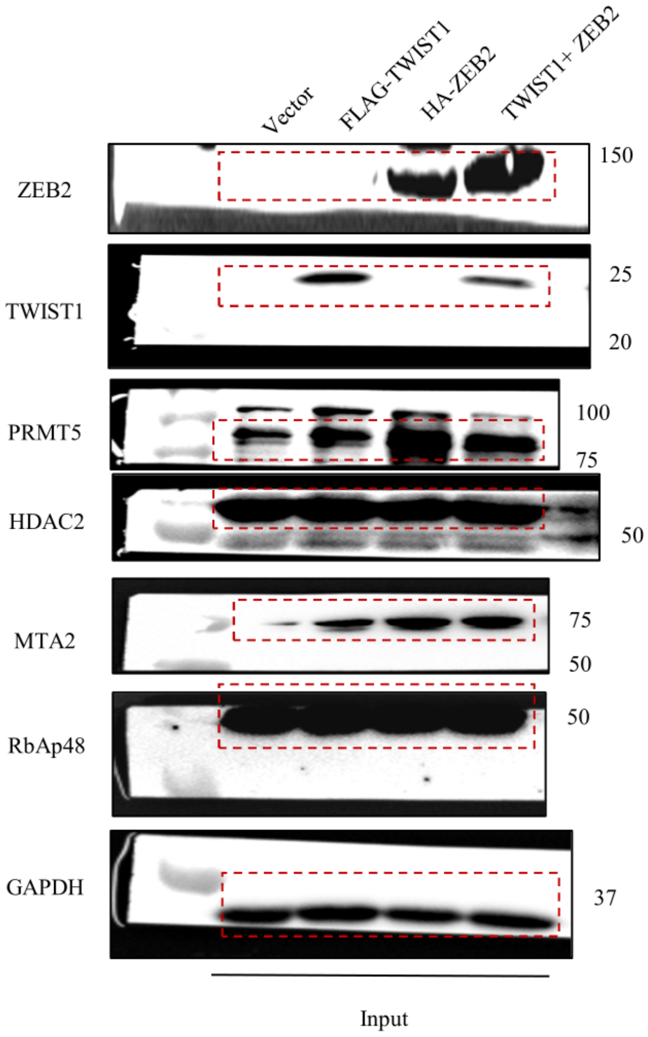

Vector
FLAG-TWIST1
HA-ZEB2
TWIST1+ ZEB2
ZEB2
150
TWIST1
25
20
PRMT5
100
75
HDAC2
50
MTA2
75
50
RbAp48
50
GAPDH
37
Input

Figure 4

C

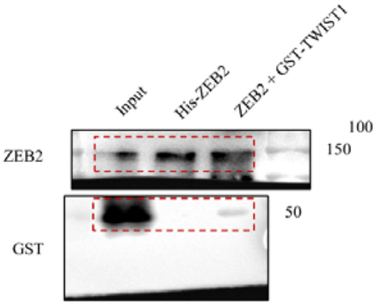
Input
His-ZEB2
ZEB2 + GST-TWIST1
ZEB2
GST
100
150
50

D

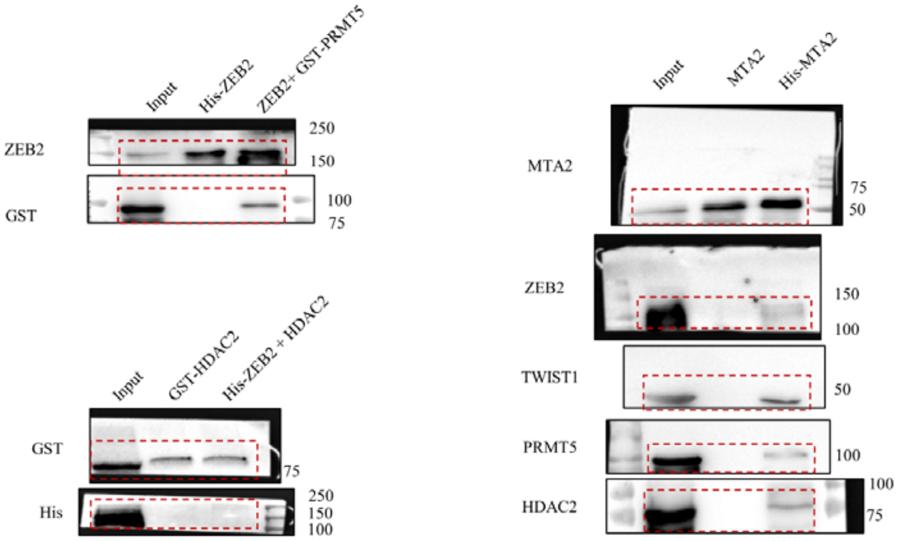
Input
His-ZEB2
ZEB2+ GST-PRMT5
ZEB2
GST
250
150
100
75
Input
GST-HDAC2
His-ZEB2 + HDAC2
GST
His
75
250
150
100
Input
MTA2
His-MTA2
MTA2
ZEB2
TWIST1
PRMT5
HDAC2
75
50
150
100
50
100
100
75

Figure 4

E

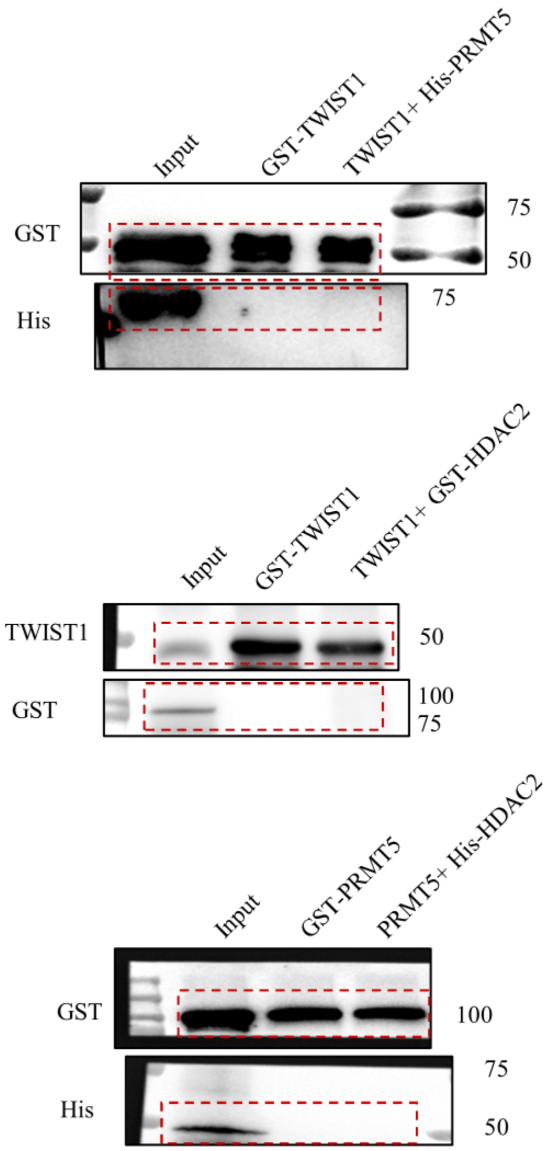

Input
GST-TWIST1
TWIST1+ His-PRMT5
GST
His
75
50
75
Input
GST-TWIST1
TWIST1+ GST-HDAC2
TWIST1
GST
50
100
75
Input
GST-PRMT5
PRMT5+ His-HDAC2
GST
His
100
75
50

Figure S2

A

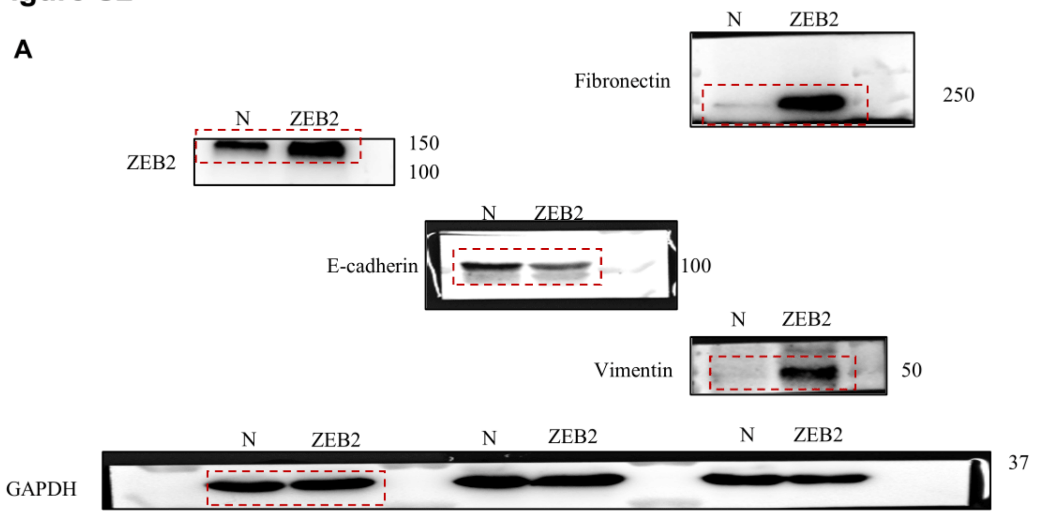
N
ZEB2
Fibronectin
250
ZEB2
150
100
E-cadherin
100
Vimentin
50
GAPDH
37

D

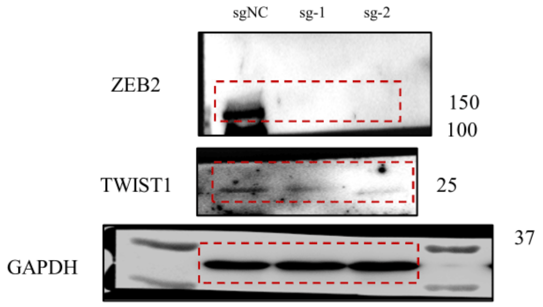
sgNC
sg-1
sg-2
ZEB2
150
100
TWIST1
25
GAPDH
37

Figure S3

B

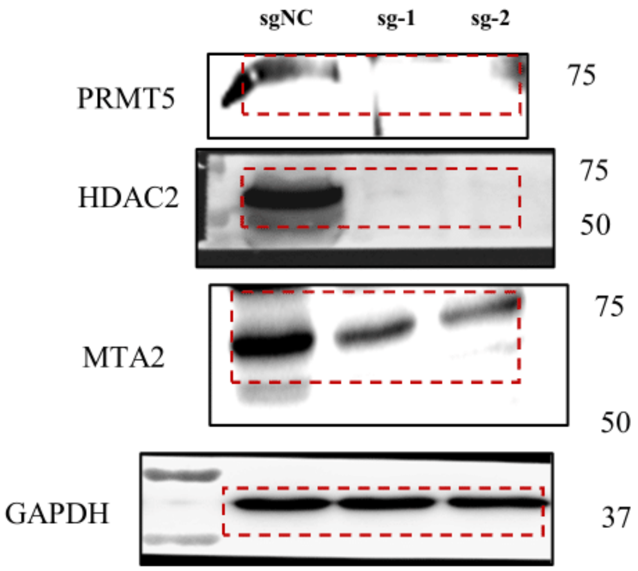

Supplement: Supplementary file 1 [file cancers-14-03426-s001.zip › cancers-1787491-supplementary.pdf]
